# Studies on Cytotoxic Constituents from the Leaves of *Elaeagnus oldhamii* Maxim. in Non-Small Cell Lung Cancer A549 Cells

**DOI:** 10.3390/molecules19079515

**Published:** 2014-07-04

**Authors:** Chi-Ren Liao, Yueh-Hsiung Kuo, Yu-Ling Ho, Ching-Ying Wang, Chang -Syun Yang, Cheng-Wen Lin, Yuan-Shiun Chang

**Affiliations:** 1 Department of Chinese Pharmaceutical Sciences and Chinese Medicine Resources, College of Pharmacy, China Medical University, Taichung 40402, Taiwan; 2 Department of Biotechnology, College of Health Science, Asia University, Wufeng, Taichung, 41354, Taiwan; 3 Department of Nursing, Hung Kuang University, Taichung 43302, Taiwan; 4 Department of Medical Laboratory Science and Biotechnology, China Medical University, Taichung 40402, Taiwan

**Keywords:** traditional herbal medicine, *Elaeagnus oldhamii* Maxim., non-small cell lung cancer A549 cells, cytotoxicity, MTT assay

## Abstract

*Elaeagnus oldhamii* Maxim. is a commonly used traditional herbal medicine. In Taiwan the leaves of *E. oldhamii* Maxim. are mainly used for treating lung disorders. Twenty five compounds were isolated from the leaves of *E. oldhamii* Maxim. in the present study. These included oleanolic acid (**1**), 3-*O*-(*Z*)-coumaroyl oleanolic acid (**2**), 3-*O*-(*E*)-coumaroyl oleanolic acid (**3**), 3-*O*-caffeoyl oleanolic acid (**4**), ursolic acid (**5**), 3-*O*-(*Z*)-coumaroyl ursolic acid (**6**), 3-*O*-(*E*)-coumaroyl ursolic acid (**7**), 3-*O*-caffeoyl ursolic acid (**8**), 3β, 13β-dihydroxyolean-11-en-28-oic acid (**9**), 3β, 13β-dihydroxyurs-11-en-28-oic acid (**10**), uvaol (**11**), betulin (**12**), lupeol (**13**), kaempferol (**14**), aromadendrin (**15**), epigallocatechin (**16**), *cis*-tiliroside (**17**), *trans*-tiliroside (**18**), isoamericanol B (**19**), *trans*-*p*-coumaric acid (**20**), protocatechuic acid (**21**), salicylic acid (**22**), *trans-*ferulic acid (**23**), syringic acid (**24**) and 3-*O*-methylgallic acid (**25**). Of the 25 isolated compounds, 21 compounds were identified for the first time in *E. oldhamii* Maxim. These included compounds **1**, **4**, **5** and **8**–**25**. These 25 compounds were evaluated for their inhibitory activity against the growth of non-small cell lung cancer A549 cells by the MTT assay, and the corresponding structure-activity relationships were discussed. Among these 25 compounds, compound **6** displayed the best activity against the A549 cell line *in vitro* (CC_50_ = 8.56 ± 0.57 μg/mL, at 48 h of MTT asssay). Furthermore, compound **2**, **4**, **8** and **18** exhibited *in vitro* cytotoxicity against the A549 cell line with the CC_50_ values of less than 20 μg/mL at 48 h of MTT asssay. These five compounds **2**, **4**, **6**, **8** and **18** exhibited better cytotoxic activity compared with cisplatin (positive control, CC_50_ value of 14.87 ± 1.94 μg/mL, at 48 h of MTT asssay). The result suggested that the five compounds might be responsible for its clinical anti-lung cancer effect.

## 1. Introduction

There are about 90 species of *Elaeagnus* around the world and nine species can be found in Taiwan [1]. Many species of *Elaeagnus* are used as folk medicinal plants, e.g., *E. umbellate* [2], *E. pungens* [3], *E. angustifolia* [4,5] and *E. multiflora* [6]. The cytotoxic activities of several species of *Elaeagnus* have been evaluated in previous studies, e.g., *E. angustifolia* [7], *E. umbellate* [8], *E. pungens* [9] and *E. glabra* [10]. Triterpenoid [11,12,13], flavonoid [14,15,16], lignanoid [17,18,19] and benzenoid [20,21,22] compounds are four of the major classes of bioactive compounds used for their anti-tumor properties. Furthermore, triterpenoid, flavonoid, lignanoid and benzenoid compounds were isolated from several species of *Elaeagnus*, e.g., *E. pungens* [23], *E. bockii* [24], *E. lanceolata*, [25,26] and *E. angustifolia* [27] in previous studies.

**Figure 1:**
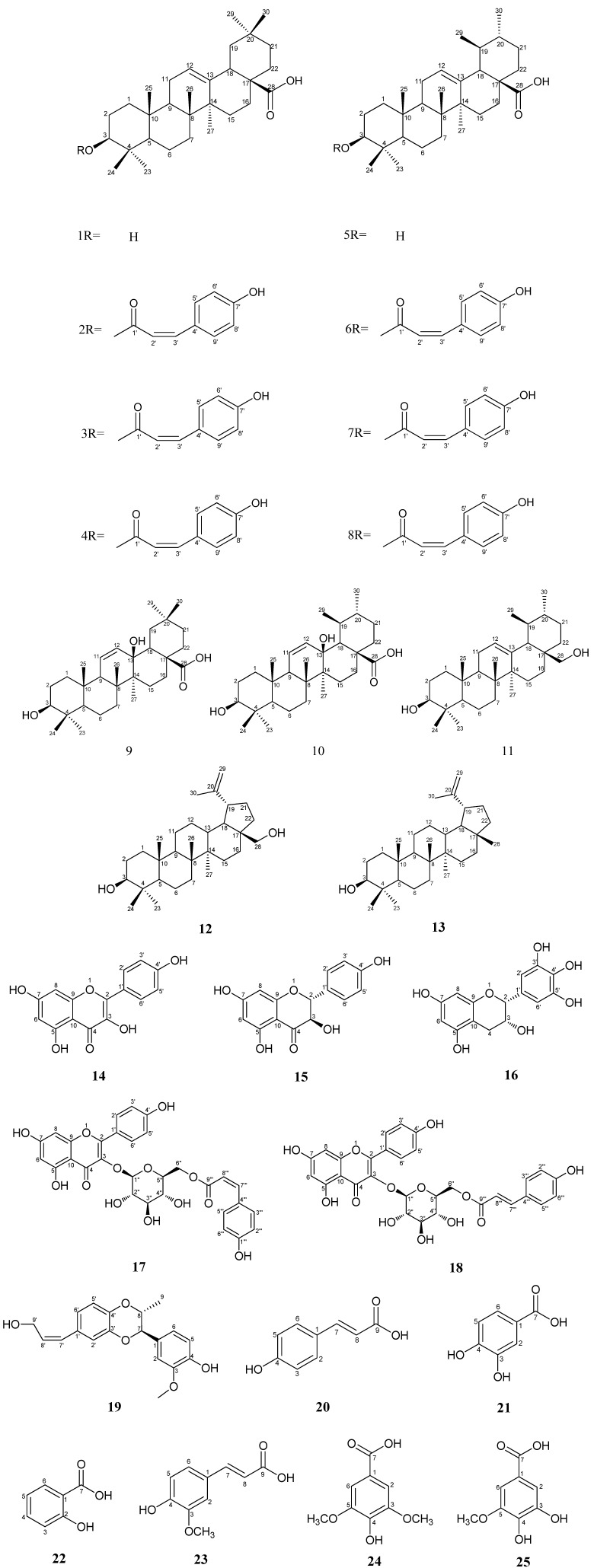
The chemical structures of compounds **1**–**25**.

Lung cancer is one of the cancers which commonly causes deaths all over the world. In addition, lung cancer is the first most lethal cancer type and the third rate of occurrence cancer in Taiwan, respectively [28,29]. Traditionally, the leaves of *E. oldhamii* Maxim. were mainly used for treating lung disorders in Taiwan, such as coughs, asthma and pulmonary abscesses. Based on our interest in the anti-lung cancer activity of this plant, EOM (methanol extract of *E. oldhamii* Maxim.) and three partitioned fractions of the methanol extract of *E. oldhamii* Maxim., including EOE (EtOAc-soluble fraction), EOB (BuOH-soluble fraction) and EOW (water-soluble fraction) had been evaluated for their inhibitory activity against the growth of non-small cell lung cancer A549 cells by MTT assays in the present study. Non-small cell lung cancer (NSCLC) accounts for 85% of all cases of lung cancer, and the A549 cells line is a kind of non-small cell lung cancer cell which was often applied to evaluate cytotoxic activity *in vitro* [30,31]. The results showed that EtOAc-soluble fraction exhibited the best activity against the A549 cell line *in vitro* (CC_50_ = 45.15 ± 1.10, at 48 h of MTT asssay). Based on our interest in the chemical components of this plant, the EtOAc-soluble fraction was further fractioned and purified. A total of 25 compounds (Figure 1) were isolated from the leaves of *E. oldhamii* Maxim. Twenty one of these 25 compounds, including compound **1**, **4**, **5** and **8**–**25**, were found for the first time in the leaves of *E. oldhamii* Maxim. Furthermore, eleven of these compounds, including **2**–**4**, **6**–**10**, **13**, **18** and **19** were evaluated for the first time regarding their cytotoxic activity in non-small cell lung cancer A549 cells by MTT assay. The isolation procedure, the ^1^H- and ^13^C-NMR spectra and the cytotoxic activity of these total 25 isolates are described herein.

## 2. Results and Discussion

The ^1^H- and ^13^C-NMR spectra of the 25 compounds isolated in present study (Table 1, Table 2, Table 3 and Section 3.4) including oleanolic acid (**1**) [32], 3-*O*-(*Z*)-coumaroyl oleanolic acid (**2**) [33], 3-*O*-(*E*)-coumaroyl oleanolic acid (**3**) [34], 3-*O*-caffeoyl oleanolic acid (**4**) [35], ursolic acid (**5**) [36], 3-*O*-(*Z*)-coumaroyl ursolic acid (**6**) [35], 3-*O*-(*E*)-coumaroyl ursolic acid (**7**) [36], 3-*O*-caffeoyl ursolic acid (**8**) [37], 3β, 13β-dihydroxyolean-11-en-28-oic acid (**9**) [37], 3β, 13β-dihydroxyurs-11-en-28-oic acid (**10**) [38], uvaol (**11**) [39], betulin (**12**) [40], lupeol (**13**) [41], kaempferol (**14**) [42], aromadendrin (**15**) [43], epigallocatechin (**16**) [44], *cis*-tiliroside (**17**) [45], *trans*-tiliroside (**18**) [46], isoamericanol B (**19**) [47], *trans*-*p*-coumaric acid (**20**) [47], protocatechuic acid (**21**) [48], salicylic acid (**22**) [49], *trans-*ferulic acid (**23**) [50], syringic acid (**24**) [51] and 3-*O*-methylgallic acid (**25**) [52] were compared with the spectral data reported in the literature and their structures were thus confirmed.

Non-small cell lung cancer (NSCLC) accounts for 85% of all lung cancer cases, and A549 cell line is one kind of non-small cell lung cancer cells which was used to evaluate cytotoxic activity *in vitro* [30,31]. Therefore, we chose the A549 cell line to evaluate cytotoxic activity of these 25 compounds. In this study, four triterpenoid compounds (compounds **2**, **4**, **6** and **8**) and one flavanoid compound (compound **18**) isolated from the crude extract displayed excellent cytotoxic activity against the A549 cell line with CC_50_ values of less than 20 μg/mL.

Oleanolic acid and ursolic acid exhibited good cytotoxic activity against the A549 cell line in previous studies [53,54,55]. However, compound **2**, **4**, **6** and **8** were oleanolic acid and ursolic acid 3-*O*- derivatives, and these four compounds showed better cytotoxic activity than oleanolic acid (compound **1**) and ursolic acid (compound **5**) in the present study. The CC_50_ values of compound **1** and compound **5** were 52.97 ± 1.22 and 154.73 ± 7.86 at 48 h of MTT assay, and compound **2**, **4**, **6** and **8** were 9.46 ± 1.50, 13.76 ± 3.45, 8.56 ± 0.57 and 15.70 ± 1.24 at 48 h of MTT assay respectively (Table 4). Furthermore, regard to function groups of these four compounds, compound **2** and **6** were oleanolic acid and ursolic acid 3-*O*-linkage to *cis*-coumaryl, and compound **4** and **8** were 3-*O*-linkage to caffeoyl. In addition, oleanolic acid and ursolic acid 3-*O*-linkage to *cis*-coumaroyl (compounds **2** and **6**) exhibited better cytotoxic activity than 3-*O*-linkage to *trans*-coumaroyl (compounds **3** and **7**). The CC_50_ value of compound **2** and **3** were 9.46 ± 1.50 and 65.21 ± 7.15, and compounds **6** and **7** were 8.56 ± 0.57 and 79.59 ± 5.77 repectively (Table 4). Therefore, we believe that 3-*O*-linkage to *cis*-coumaroyl and caffeoyl of oleanolic acid and ursolic acid were two active functional groups responsible for the cytotoxic activity against the growth of non-small cell lung cancer A549 cells according to the MTT assay.

Kaempferol was investigated for its cytotoxic activity against the A549 cell line in a previous study [56]. Compound **17** was a kaempferol derivative and exhibited better cytotoxic activity than kaempferol (compound **14**) in present study. The CC_50_ value of compound **14** was 113.48 ± 5.32, and that of compound **17** was 18.82 ± 3.64 at 48 h of MTT assay, respectively (Table 4). Furthermore, with regard to the functional groups of compound **17**, it has a kaempferol 3-*O*-linkage to d-glucose and a 6''-*O*-linkage to a *cis*-coumaroyl moiety. In addition, the kaempferol 6''-*O*-linkage to a *cis*-coumaroyl group (compound **17**) exhibited better cytotoxic activity than a 6''-*O*-linkage to a *trans*-coumaroyl group (compound **18**). The CC_50_ value of compound **17** was 18.82 ± 3.64, and that of compound **18** was 144.74 ± 5.37 at 48 h MTT assay, respectively (Table 4). Therefore, we also believe that in this case the 3-*O*-linkage to d-glucose and 6''-*O*-linkage to the *cis*-coumaroyl were the active functional groups that provide better cytotoxic activity against the growth of non-small cell lung cancer A549 cells according to the MTT assay.

**Table 1:** ^13^C-NMR spectroscopic data (500 MHz) of compounds **1**–**13**.

Position	1 *^d^*		2 *^b^*		3 *^c^*		4 *^d^*		5 *^d^*		6 *^b^*		
1	39.4		37.2		39.3		38.6		39.4		38.0		
2	28.5		23.1		24.1		24.1		28.4		28.2		
3	78.5		81.4		85.7		80.9		78.5		81.4		
4	39.8		38.0		39.4		38.7		39.8		38.5		
5	56.2		55.5		56.6		56.1		56.2		55.5		
6	19.2		18.4		19.6		19.0		19.1		18.4		
7	33.7		32.7		33.7		33.5		33.9		33.0		
8	40.2		39.5		40.8		40.2		40.3		39.7		
9	48.6		47.7		49.1		48.4		48.4		48.2		
10	37.8		38.3		39.4		37.7		37.8		37.1		
11	24.2		23.7		24.8		24.6		34.0		23.5		
12	123.0		122.8		123.5		122.8		126.0		126.0		
13	145.2		143.8		146.4		144.4		139.6		138.2		
14	42.6		41.8		43.1		42.6		42.9		42.1		
15	28.7		27.9		28.9		28.8		29.0		28.3		
16	24.2		22.9		24.1		24.2		25.3		24.2		
17	47.1		46.8		47.8		47.2		48.4		47.6		
18	42.4		41.1		42.9		42.5		53.9		52.7		
19	46.9		46.1		47.4		47.0		39.7		39.2		
20	31.4		30.9		29.4		31.5		39.7		39.0		
21	34.7		34.0		35.0		34.8		31.4		30.8		
22	33.6		32.7		31.8		32.6		37.6		37.0		
23	29.2		28.3		28.9		28.7		29.2		29.9		
24	17.0		15.6		17.9		17.9		16.9		17.0		
25	16.0		16.9		17.2		15.9		16.0		15.8		
26	17.9		17.4		18.4		17.6		17.8		17.2		
27	26.6		26.1		26.6		26.7		24.2		23.8		
28	180.6		184.3		182.0		180.9		180.2		184.1		
29	33.7		33.3		33.9		33.8		17.8		17.3		
30	24.1		23.6		24.2		23.8		21.8		21.4		
1'	-		167.0		169.8		167.8		-		167.0		
2'	-		117.7		116.0		116.4		-		117.7		
3'	-		143.7		145.5		145.4		-		143.8		
4'	-		127.5		127.5		127.4		-		127.5		
5'	-		132.4		131.2		116.0		-		132.4		
6'	-		115.3		117.0		146.2		-		115.3		
7'	-		157.1		161.3		149.9		-		157.2		
8'	-		115.3		117.0		117.4		-		115.3		
9'	-		132.4		131.2		122.5		-		132.4		
**Position**	**7 *^c^***	**8 *^c^***		**9 *^b^***		**10 *^b^***		**11 *^b^***		**12 *^b^***		**13 *^b^***	
1	39.3	37.9		37.5		38.4		39.0		38.9		38.9	
2	29.3	24.5		23.8		23.0		27.5		27.6		29.9	
3	85.7	81.0		79.0		79.1		79.2		79.2		79.2	
4	40.6	37.6		38.5		39.1		38.2		38.9		39.1	
5	56.6	56.2		53.4		55.0		55.4		55.5		55.5	
6	19.6	19.8		18.2		17.9		18.5		18.5		18.6	
7	34.3	33.5		29.9		29.9		33.0		34.5		34.5	
8	41.0	40.3		41.6		41.9		40.2		39.8		41.1	
9	50.0	47.6		50.7		53.3		47.9		50.6		50.7	
10	38.2	36.6		34.6		36.6		37.1		37.5		37.4	
11	24.3	24.2		136.1		133.7		23.6		21.0		21.2	
12	126.8	123.0		127.1		129.1		125.3		25.4		23.6	
13	139.9	145.1		90.1		89.9		138.9		37.4		38.3	
14	43.5	42.7		41.8		42.2		42.3		41.1		43.1	
15	29.4	28.6		27.4		27.2		26.2		27.3		27.7	
16	25.4	26.4		25.6		25.8		23.6		29.4		35.8	
17	49.3	46.9		44.2		45.3		37.1		48.0		42.2	
18	54.4	48.5		55.0		60.8		54.2		42.9		48.5	
19	40.8	39.0		36.6		38.5		39.6		49.0		48.2	
20	40.6	38.9		39.2		40.5		39.6		150.7		151.2	
21	31.9	31.4		31.4		31.5		30.8		30.0		30.1	
22	38.2	36.9		31.7		31.6		35.4		34.2		40.2	
23	31.0	28.5		28.0		28.0		28.3		28.2		28.2	
24	17.8	17.1		15.1		15.1		15.9		15.6		14.8	
25	17.4	15.9		17.9		16.3		15.8		16.3		16.3	
26	17.9	17.7		19.2		19.1		17.0		16.2		16.2	
27	24.6	24.0		18.5		18.1		23.5		14.7		15.6	
28	181.8	179.1		180.2		180.1		70.1		60.8		18.2	
29	18.5	17.7		33.5		18.0		17.6		109.9		109.5	
30	21.7	23.9		27.2		19.4		21.5		19.3		19.5	
1'	169.8	167.3		-		-		-		-		-	
2'	116.0	116.2		-		-		-		-		-	
3'	146.4	145.5		-		-		-		-		-	
4'	127.5	127.2		-		-		-		-		-	
5'	131.2	115.2		-		-		-		-		-	
6'	117.0	146.5		-		-		-		-		-	
7'	161.3	148.9		-		-		-		-		-	
8'	117.0	116.4		-		-		-		-		-	
9'	131.2	122.5		-		-		-		-		-	

*^b^* In chloroform-*d*; *^c^* In methanol-*d*_4_; *^d^* In pyridine-*d*_5_.

**Table 2:** ^13^C-NMR spectroscopic data (500 MHz) of compounds **14**–**18**.

Position	14 *^c^*		15 *^c^*		16 *^c^*		17 *^a^*		18 *^b^*		
1	-		-		-		-		-		
2	148.2		85.1		79.5		158.8		159.5		
3	137.3		73.8		67.1		135.3		135.4		
4	177.5		198.5		30.7		179.1		179.4		
5	162.7		165.5		157.6		158.0		158.5		
6	99.4		97.6		96.2		99.8		100.1		
7	165.8		169.4		157.6		165.4		166.0		
8	94.6		96.6		95.8		94.8		95.0		
9	158.4		164.7		157.2		162.9		163.1		
10	104.7		101.9		99.9		105.5		105.7		
1'	123.9		129.5		131.6		122.4		122.9	
2'	130.8		130.5		107.0		132.4		132.4	
3'	116.5		116.3		146.2		116.1		116.2	
4'	140.7		159.4		133.0		161.0		161.5	
5'	116.5		116.3		146.2		116.1		116.2	
6'	130.8		130.5		107.0		132.4		132.4	
1''	-		-		-		104.5		104.2	
2''	-		-		-		75.5		75.9	
3''	-		-		-		71.1		71.9	
4''	-		-		-		75.3		75.9	
5''	-		-		-		78.1		78.1	
6''	-		-		-		63.9		64.5	
1'''	-		-		-		127.3		127.2	
2'''	-		-		-		133.8		133.9	
3'''	-		-		-		115.7		115.9	
4'''	-		-		-		159.7		161.3	
5'''	-		-		-		115.7		115.9	
6'''	-		-		-		133.8		133.9	
7'''	-		-		-		144.5		146.7	
8'''	-		-		-		116.3		116.9	
9'''	-		-		-		166.3		169.0	

*^a^* In acetone-*d*_6_; *^b^* In chloroform-*d*; *^c^* In methanol-*d*_4_.

**Table 3:** ^13^C-NMR spectroscopic data (500 MHz) of compounds **19**–**25**.

Position	19 *^a^*		20 *^c^*		21 *^c^*		22 *^a^*		23 *^c^*		24 *^c^*		25 *^c^*		
1	129.4		127.4		123.2		114.3		128.1		120.6		122.4		
2	111.3		131.2		117.5		163.3		111.8		106.8		106.5		
3	148.5		116.9		145.7		118.3		150.6		147.4		146.3		
4	147.6		161.3		150.8		136.6		149.5		140.1		140.5		
5	117.9		116.9		115.8		120.2		116.6		147.4		149.2		
6	120.3		131.2		123.7		131.7		124.0		106.8		112.5		
7	78.1		146.7		167.8		173.8		146.7		167.2		170.5		
8	73.8		116.0		-		-		116.6		-		-		
9	13.7		171.3		-		-		171.5		-		-		
1'	56.5	-		-		-		-		-		-	
2'	132.8		-		-		-		-		-		-	
3'	118.3		-		-		-		-		-		-	
4'	143.9		-		-		-		-		-		-	
5'	142.6		-		-		-		-		-		-	
6'	115.9		-		-		-		-		-		-	
7'	123.5		-		-		-		-		-		-	
8'	129.6		-		-		-		-		-		-	
9'	131.5		-		-		-		-		-		-	
OCH_3_	59.8								56.6		55.9		56.8	

*^a^* In acetone-*d*_6_; *^c^* In methanol-*d*_4_.

**Table 4:** Cytotoxicities of compound **1**–**25** from *E. oldhamii* Maxim on A549 cell line.

Sample Code	Compound Name	CC_50_ (μg/mL)	
		24 h	48 h
Positive control	Cisplatin	132.05 ± 1.46	14.87 ± 1.94
**1**	Oleanolic acid	64.86 ± 3.90	52.97 ± 1.22
**2**	3-*O*-(*Z*)-coumaroyl oleanolic acid	9.23 ± 1.73	9.46 ± 1.50
**3**	3-*O*-(*E*)-coumaroyl oleanolic acid	135.12 ± 3.08	65.21 ± 7.15
**4**	3-*O*-caffeoyl oleanolic acid	12.35 ± 1.52	13.76 ± 3.45
**5**	Ursolic acid	153.37 ± 3.64	154.73 ± 7.86
**6**	3-*O*-(*Z*)-coumaroyl ursolic acid	13.06 ± 2.26	8.56 ± 0.57
**7**	3-*O*-(*E*)-coumaroyl ursolic acid	121.53 ± 9.68	79.59 ± 5.77
**8**	3-*O*-caffeoyl ursolic acid	157.98 ± 5.02	15.70 ± 1.24
**9**	3β, 13β-Dihydroxyolean-11-en-28-oic acid	92.71 ± 3.89	70.61 ± 8.27
**10**	3β, 13β-Dihydroxyurs-11-en-28-oic acid	176.81 ± 6.33	156.20 ± 8.10
**11**	Uvaol	169.45 ± 9.47	163.36 ± 7.57
**12**	Betulin	>500	>500
**13**	Lupeol	142.70 ± 4.99	119.30 ± 5.28
**14**	Kaempferol	129.15 ± 5.16	113.48 ± 5.32
**15**	Aromadendrin	76.66 ± 4.35	55.67 ± 2.37
**16**	Epigallocatechin	193.77 ± 3.98	118.92 ± 4.25
**17**	*cis*-Tiliroside	68.05 ± 1.46	18.82 ± 3.64
**18**	*trans*-Tiliroside	149.90 ± 4.14	144.74 ± 5.37
**19**	Isoamericanol B	>500	126.97 ± 4.83
**20**	*trans*-*p*-Coumaric acid	>500	>500
**21**	Protocatechuic acid	>500	>500
**22**	Salicylic acid	>500	103.64 ± 4.59
**23**	*trans-*Ferulic acid	78.09 ± 5.78	60.42 ± 5.00
**24**	Syringic acid	73.41 ± 2.92	77.66 ± 4.04
**25**	3-*O*-methylgallic acid	>500	180.60 ± 8.58
MOM		84.67 ± 0.37	89.98 ± 7.03
MOE		52.78 ± 0.78	45.15 ± 1.10
MOB		192.46 ± 6.54	166.38 ± 12.65
MOW		100.91 ± 1.01	136.47 ± 3.96

## 3. Experimental

### 3.1. General

The 1D- and 2D-NMR spectra were recorded with a Bruker DRX-500 FT-NMR (Taichung, Taiwan) spectrometer. HPLC chromatograms were obtained with an LC-6A instrument and an IOTA-2 RI-detector (Shimadzu, Kyoto, Japan). Semi-preparative NP-HPLC column chromatography was performed using MonoChrom Si gel (250 mm × 10 mm i.d., 5 μm, MetaChem, Torrance, CA, USA), and semi-preparative RP-HPLC column chromatography was performed using a Discovery^®^ C18 column (250 mm × 10 mm i.d., 5 μm, Supelco, Bellefonte, PA, USA), TLC was performed with aluminum pre-coated Si plates (Merck, Darmstadt, Germany), Silica gel (63–200 μm) and Sephadex^®^ LH-20 was (25–100 μm) were purchased from Merck. A multiscan microplate reader (VersaMax ELISA Microplate Reader, Taipei, Taiwan) was used for the MTT assays. The solvents used in the study for open column isolation (Sephadex LH 20 and silica gel column), such as *n*-hexane, chloroform, ethyl acetate, acetone and methanol were ACS grade and purchased from Merck. HPLC grade *n*-hexane, chloroform, ethyl acetate, acetone, methanol, acetonitrile and ammonium acetate were purchased from Merck. De-ionised water was prepared by an Elix 10/Milli-Q Gradient (Millipore, Billerica, MA, USA). Acetone-*d*_6_, chloroform-*d*, methanol-*d*_4_ and pyridine-*d*_5_ for NMR measurements were purchased from Merck. MTT (3-[4,5-dimethylthiazol-2-yl]-2,5-diphenyltetrazolium bromide) and Dulbecco’s modified Eagle’s medium (DMEM) were purchased from Sigma Chemical Co. (St. Louis, MO, USA). FBS (Fetal bovine serum) and penicillin/streptomycin were purchased from Gibco-BRL (New York, NY, USA). The A549 cell line was purchased from the Food Industry Research and Development Institute (Hsinchu, Taiwan).

### 3.2. Plant Material

The leaves of *E. oldhamii* Maxim. as described in Flora of Taiwan [1] were purchased from the Jin-Shun Shen herbal garden (Nantou, Taiwan) in October 2012. The plant materials were also identified by Yuan-Shiun Chang, Department of Chinese Pharmaceutical Sciences and Chinese Medicine Resources, College of Pharmacy, China Medical University, Taichung, Taiwan. A plant specimen has been deposited in the Department of Chinese Pharmaceutical Sciences and Chinese Medicine Resources with voucher specimen number CMU-CMR-2013-EO-1.

### 3.3. Extraction and Isolation

The materials were totally dried in air under dark. Dried leaves of *E. oldhamii* Maxim. (10.0 kg) were cut into small pieces and soaked in 70 L of methanol for 7 days. The extraction was repeated three times. The extracts were combined and filtered through filter paper and concentrated under reduced pressure to give a dried extract (801.0 g). The dried extract was suspended in H_2_O (2 L) and extracted with ethyl acetate (2 L, five times). The resulting ethyl acetate extract was concentrated to yield 302.5 g of a brown-green thick oil. The fraction was further purified on 2.2 kg of silica gel with particle size 0.063–0.200 mm on a column with 10 cm internal diameter and 50 cm length using a gradient of increasing polarity from total *n*-hexane to total ethyl acetate as mobile phase and was separated into 20 fractions. Fraction 1 (0.52 g) was isolated from the 100% *n*-hexane mobile phase. Fraction 2 (12.28 g) was isolated from the 1:19 *n*-hexane/ethyl acetate mobile phase. Fraction 3 (18.95 g) was isolated from the 1:9 *n*-hexane/ethyl acetate mobile phase. Fractions 4 (10.28 g), 5 (11.41 g), 6 (10.55 g), 7 (15.20 g) and 8 (8.85 g) were isolated from the 8:2 *n*-hexane/ethyl acetate mobile phase. Fractions 9 (16.69 g) and 10 (13.44 g) were isolated from the 7:3 *n*-hexane/ethyl acetate mobile phase. Fractions 11 (19.18 g), 12 (11.74 g), 13 (7.84 g) and 14 (11.40 g) were isolated from the 5:5 *n*-hexane/ethyl acetate mobile phase. Fractions 15 (6.02 g), 16 (14.38 g) and 17 (6.01 g) were isolated from the 3:7 *n*-hexane/ethyl acetate mobile phase. Fractions 18 (19.91 g), 19 (12.57 g) and 20 (6.13 g) were isolated from the 100% ethyl acetate mobile phase. The ethyl acetate extract remaining on the column was eluted by methanol (28.92 g). The weight of the 20 fractions and the fraction which was eluted by methanol was 261.94 g, so the recovery rate was 86.59%.

Twenty five compounds were isolated in fractions 8, 14 and 15 respectively. Compounds **1** (645.0 mg), **2** (32.1 mg), **3** (22.5 mg), **5** (655.2 mg), **6** (52.0 mg), **7** (15.3 mg), **9** (10.2 mg), **10** (8.5 mg), **11** (11.5 mg), **12** (14.0 mg) and **13** (12.8 mg) were isolated from fraction 8. Compounds **4** (8.8 mg), **8** (13.7 mg), **14** (22.6 mg), **15** (2.7 mg), **16** (11.7 mg), **19** (11.8 mg), **20** (22.0 mg), **21** (45.2 mg), **22** (8.2 mg), **23** (5.8 mg), **24** (14.0 mg) and **25** (8.3 mg) were isolated from fraction 14. Compounds **17** (32.8 mg), and **18** (10.3 mg) were isolated from fraction 15. The isolation procedure of these 25 compounds was described below.

Fraction 8 (8.85 g) was washed with *n*-hexane and acetone to obtain a mixture of compounds **1** and **5** (total weight equivalent to 1,306.5 mg). These two compounds were separated by semi-preparative RP-HPLC (acetonitrile/0.1% ammonium acetate solution = 9:1, v/v) to afford pure compounds **1** and **5**. The remaining fraction 8 (8.14 g) was separated by silica gel column chromatography (*n*-hexane/ethyl acetate = 8:2, v/v) to give four subfractions (subfraction 8–1 to 8–4). Compounds **2**, **3**, **6** and **7** were obtained from subfraction 8–1 through semi-preparative NP-HPLC (*n*-hexane/acetone = 8:2, v/v). Compounds **9** and **10** were obtained from subfraction 8–2 through semi-preparative NP-HPLC (*n*-hexane/acetone = 8:2, v/v) and semi-preparative RP-HPLC (acetonitrile/water = 19:1, v/v). Compound **11** was obtained from subfraction 8–3 through semi-preparative NP-HPLC (*n*-hexane/acetone = 8:2, v/v). Compounds **12** and **13** were obtained from subfraction 8–4 through semi-preparative NP-HPLC (*n*-hexane/acetone = 8:2, v/v).

Fraction 14 (11.40 g) was separated by Sephadex LH 20 column chromatography (chloroform/methanol = 3:7, v/v) to give six subfractions (subfraction 14–1 to 14–6). Compounds **4**, **8**, **23** and **25** were obtained from subfraction 14–2 through semi-preparative NP-HPLC (*n*-hexane/acetone = 1:1, v/v) and semi-preparative RP-HPLC (acetonitrile/water = 8:2, v/v). Compound **19** was obtained from subfraction 14–2 through semi-preparative NP-HPLC (*n*-hexane/acetone = 1:1, v/v) and NP-HPLC (*n*-hexane/ethyl acetate = 1:1, v/v). Compound **22** was obtained from subfraction 14–3 through semi-preparative NP-HPLC (*n*-hexane/acetone = 1:1, v/v). Compound **24** was obtained from subfraction 14–3 through semi-preparative NP-HPLC (*n*-hexane/acetone = 1:1, v/v) and semi-preparative RP-HPLC (*n*-hexane/ethyl acetate = 1:1, v/v). Compound **20** was obtained from subfraction 14–4 through semi-preparative NP-HPLC (*n*-hexane/acetone = 3:2, v/v) and NP-HPLC (*n*-hexane/ethyl acetate = 52:48, v/v). Compounds **14**, **15**, and **21** were obtained from subfraction 14–5 through semi-preparative NP-HPLC (*n*-hexane/acetone = 8:2, v/v). Compound **16** was obtained from subfraction 14–5 through semi-preparative NP-HPLC (*n*-hexane/acetone = 8:2, v/v) and semi-preparative NP-HPLC (*n*-hexane/ethyl acetate = 2:3, v/v). Fraction 15 (6.02 g) was separated by Sephadex LH 20 column chromatography (chloroform/methanol = 3:7, v/v) to give four subfractions (subfraction 15–1 to 15–4). Compounds **17** and **18** were obtained from subfraction 15–3 through semi-preparative NP-HPLC (*n*-hexane/acetone = 2:3, v/v) and semi-preparative NP-HPLC (*n*-hexane/acetone = 1:2, v/v).

### 3.4. Spectroscopic Data

*Oleanolic acid* (**1**). White powder, ^1^H-NMR (500 MHz, C_5_D_5_N): δ 5.52 (1H, *br s*, H-12), δ 3.46 (1H, *dd*, *J* = 10.5, 5.5 Hz, H-3), δ 3.33 (1H, *dd*, *J* = 13.8, 3.7 Hz, H-18), δ 1.30 (3H, *s*), δ 1.26 (3H, *s*), δ 1.04 (6H, *s*), δ 1.03 (3H, *s*), δ 0.97 (3H, *s*), δ 0.91 (3H, *s*), δ 0.88 (1H, *d*, *J* = 12.0 Hz, H-5). The above data were identical to the literature data [32].

*3-O-(Z)-Coumaroyl oleanolic acid* (**2**). White powder, ^1^H-NMR (500 MHz, C_5_D_5_N): δ 8.12 (2H, *d*, *J* = 8.6 Hz, H-5' and H-9'), δ 7.21 (2H, *d*, *J* = 8.6 Hz, H-6' and H-8'), δ 7.01 (1H, *d*, *J* = 12.9 Hz, H-3'), δ 6.05 (1H, *d*, *J* = 12.9 Hz, H-2'), δ 5.50 (1H, *br s*, H-12), δ 4.82 (1H, *dd*, *J* = 11.8 , 4.6 Hz, H-3), δ 1.28 (3H, *s*), δ 1.03 (3H, *s*), δ 1.00 (3H, *s*), δ 0.98 (3H, *s*), δ 0.97 (3H, *s*), δ 0.90 (3H, *s*), δ 0.85 (3H, *s*). The above data were identical to the literature data [33].

*3-O-(E)-Coumaroyl oleanolic acid* (**3**). White powder, ^1^H-NMR (500 MHz, C_5_D_5_N): δ 8.03 (1H, *d*, *J* = 15.9 Hz, H-3'), δ 7.68 (2H, *d*, *J* = 8.6 Hz, H-5' and H-9'), δ 7.19 (2H, *d*, *J* = 8.6 Hz, H-6' and H-8'), δ 6.71 (1H, *d*, *J* = 15.9 Hz, H-2'), δ 5.49 (1H, *br s*, H-12), δ 4.90 (1H, *br d*, *J* = 9.8 Hz, H-3), δ 1.29 (3H, *s*), δ 1.03 (3H, *s*), δ 1.02 (3H, *s*), δ 0.98 (6H, *s*), δ 0.97 (3H, *s*), δ 0.87 (3H, *s*). The above data were identical to the literature data [33].

*3-O-Caffeoyl oleanolic acid* (**4**). White powder, ^1^H-NMR (500 MHz, CD_3_OD): δ 7.53 (1H, *d*, *J* = 15.8 Hz, H-3'), δ 7.03 (1H, *s*, H-5'), δ 6.93 (1H, *d*, *J* = 8.1 Hz, H-9'), δ 6.77 (1H, *d*, *J* = 8.1 Hz, H-8'), δ 6.23 (1H, *d*, *J* = 15.8 Hz, H-2'), δ 5.24 (1H, *br s*, H-12), δ 4.56 (1H, *br d*, *J* = 10.0 Hz, H-3), δ 1.17 (3H, *s*), δ 1.00 (3H, *s*), δ 0.96 (3H, *s*), δ 0.94 (3H, *s*), δ 0.90 (6H, *s*), δ 0.83 (3H, *s*). The above data were identical to the literature data [34].

*Ursolic acid* (**5**). White powder, ^1^H-NMR (500 MHz, C_5_D_5_N): δ 5.49 (1H, *br s*, H-12), δ 3.45 (1H, *dd*, *J* = 9.7, 6.4 Hz, H-3), δ 2.63 (1H, *d*, *J* = 11.4 Hz, H-18), 1.24 (3H, *s*), 1.23 (3H, *s*), 1.05(3H, *s*), δ 1.02 (3H, *s*), δ 1.00 (1H, *d*, *J* = 6.5 Hz), δ 0.96 (1H, *d*, *J* = 6.2 Hz), δ 0.89 (3H, *s*), δ 0.86 (1H, *d*, *J* = 11.5 Hz, H-5). The above data were identical to the literature data [32].

*3-O-(Z)-coumaroyl ursolic acid* (**6**) White powder, ^1^H-NMR (500 MHz, CDCl_3_): δ 7.59 (2H, *d*, *J* = 8.1 Hz, H-5' and H-9'), δ 6.76 (2H, *d*, *J* = 8.1 Hz, H-6' and H-8'), δ 6.84 (1H, *d*, *J* = 12.8 Hz, H-3'), δ 5.83 (1H, *d*, *J* = 12.8 Hz, H-2'), δ 5.23 (1H, *br s*, H-12), δ 4.56 (1H, *br d*, *J* = 9.3 Hz, H-3), δ 1.08 (3H, *s*), δ 0.95 (6H, *s*), δ 0.87 (3H, *s*), δ 0.86 (3H, *s*), δ 0.82 (3H, *s*), δ 0.76 (3H, *s*). The above data were identical to the literature data [35].

*3-O-(E)-Coumaroyl ursolic acid* (**7**). White powder, ^1^H-NMR (500 MHz, CD_3_OD): δ 7.62 (1H, *d*, *J* = 15.9 Hz, H-3'), δ 7.45 (2H, *d*, *J* = 8.6 Hz, H-5' and H-9'), δ 6.80 (2H, *d*, *J* = 8.6 Hz, H-6' and H-8'), δ 6.38 (1H, *d*, *J* = 15.9 Hz, H-2'), δ 5.24 (1H, *br s*, H-12), δ 4.63 (1H, *br d*, *J* = 9.9 Hz, H-3), δ 1.14 (3H, *s*), δ 1.06 (3H, *s*), δ 0.97 (3H, *s*), δ 0.94 (3H, *s*), δ 0.89 (3H, *s*), δ 0.88 (3H, *s*), δ 0.86 (3H, *s*). The above data were identical to the literature data [35].

*3-O-Caffeoyl ursolic acid* (**8**). White powder, ^1^H-NMR (500 MHz, CD_3_OD): δ 7.53 (1H, *d*, *J* = 15.9 Hz, H-3'), δ 7.16 (1H, *s*, H-5'), δ 7.03 (1H, *d*, *J* = 8.2 Hz, H-9'), δ 6.85 (1H, *d*, *J* = 8.2 Hz, H-8'), δ 6.29 (1H, *d*, *J* = 15.9 Hz, H-2'), δ 5.25 (1H, *br s*, H-12), δ 4.75 (1H, *dd*, *J* = 11.4 , 4.5 Hz, H-3), δ 1.20 (3H, *s*), δ 1.00 (3H, *s*), δ 0.95 (3H, *s*), δ 0.94 (3H, *s*), δ 0.92 (3H, *s*), δ 0.90 (3H, *s*), δ 0.82 (3H, *s*). The above data were identical to the literature data [36].

*3β, 13β-Dihydroxyolean-11-en-28-oic acid* (**9**). White powder, ^1^H-NMR (500 MHz, CDCl_3_): δ 6.04 (1H, *d*, *J* = 10.2 Hz, H-12), δ 5.41 (1H, *dd*, *J* = 10.2, 2.4 Hz, H-11), δ 3.22 (1H, *dd*, *J* = 11.4, 4.3 Hz, H-3), δ 1.25 (3H, *s*), δ 1.06 (3H, *s*), δ 0.98 (3H, *s*), δ 0.97 (3H, *s*), δ 0.91 (3H, *s*), δ 0.88 (3H, *s*), δ 0.78 (3H, *s*). The above data were identical to the literature data [37].

*3β, 13β-Dihydroxyurs-11-en-28-oic acid* (**10**). White powder, ^1^H-NMR (500 MHz, CDCl_3_): δ 5.96 (1H, *d*, *J* = 10.2 Hz, H-12), δ 5.25 (1H, *br d*, *J* = 10.2, H-11), δ 3.22 (1H, *dd*, *J* = 11.6, 4.6 Hz, H-3), δ 1.16 (3H, *s*), δ 1.00 (3H, *d*, *J* = 6.3), δ 0.99 (3H, *s*), δ 0.94 (3H, *s*), δ 0.94 (3H, *d*, *J* = 6.3), δ 0.91 (3H, *s*), δ 0.78 (3H, *s*).The above data were identical to the literature data [38].

*Uvaol* (**11**). White powder, ^1^H-NMR (500 MHz, CDCl_3_): δ 5.12 (1H, *t*, *J* = 3.5 Hz, H-12), δ 3.54 (1H, *d*, *J* = 11.0 Hz, H-28β), δ 3.20 (1H, *dd*, *J* = 11.2, 5.0 Hz, H-3), δ 3.18 (1H, *d*, *J* = 11.0 Hz, H-28α), δ 1.09 (3H, *s*), δ 0.99 (3H, *s*), δ 0.94 (3H, *s*), δ 0.93 (3H, *d*, *J* = 5.8 Hz), δ 0.80 (3H, *d*, *J* = 5.8 Hz), δ 0.78 (3H, *s*), δ 0.72 (3H, *d*, *J* = 11.2 Hz, H-5). The above data were identical to the literature data [39].

*Betulin* (**12**). White powder, ^1^H-NMR (500 MHz, CDCl_3_): δ 4.68 (1H, *br s*, H-29β), δ 4.58 (1H, *br s*, H-29α), δ 3.79 (1H, *d*, *J* = 10.8 Hz, H-28β), δ 3.32 (1H, *d*, *J* = 10.8 Hz, H-28α), δ 3.20 (1H, *dd*, *J* = 11.4, 4.6 Hz, H-3), δ 1.67 (3H, *s*), δ 1.01 (6H, *s*), δ 0.98 (3H, *s*), δ 0.96 (3H, *s*), δ 0.82 (3H, *s*), δ 0.75 (3H, *s*). The above data were identical to the literature data [40].

*Lupeol* (**13**). White powder, ^1^H-NMR (500 MHz, CDCl_3_): δ 4.68 (1H, *br s*, H-29β), δ 4.56 (1H, *br s*, H-29α), δ 3.18 (1H, *dd*, *J* = 11.4, 4.9 Hz, H-3), δ 1.68 (3H, *s*), δ 1.02 (3H, *s*), δ 0.96 (3H, *s*), δ 0.94 (3H, *s*), δ 0.83 (3H, *s*), δ 0.79 (3H, *s*), δ 0.76 (3H, *s*). The above data were identical to the literature data [41].

*Kaempferol* (**14**). Yellowish powder, ^1^H-NMR (500 MHz, CD_3_OD): δ 8.06 (2H, *d*, *J* = 8.8 Hz, H-2' and H-6'), δ 6.88 (2H, *d*, *J* = 8.8 Hz, H-3' and H-5'), δ 6.37 (1H, *d*, *J* = 1.7 Hz, H-8), δ 6.16 (1H, *d*, *J* = 1.7 Hz, H-6). The above data were identical to the literature data [42].

*Aromadendrin* (**15**). White powder, ^1^H-NMR (500 MHz, CD_3_OD): δ 11.71 (1H, *br s*, OH), δ 7.41 (2H, *d*, *J* = 8.5 Hz, H-2' and H-6'), δ 6.89 (2H, *d*, *J* = 8.5 Hz, H-3' and H-5'), δ 5.99 (1H, *d*, *J* = 1.9 Hz, H-8), δ 5.94 (1H, *d*, *J* = 1.9 Hz, H-6), δ 5.08 (1H, *d*, *J* = 11.5 Hz, H-2), δ 5.65 (1H, *d*, *J* = 11.5 Hz, H-3). The above data were identical to the literature data [43].

*Epigallocatechin* (**16**). White powder, ^1^H-NMR (500 MHz, CD_3_OD): δ 8.17 (1H, *br s*, OH), δ 8.01 (1H, *br s*, OH), δ 7.92 (2H, *br s*, OH), δ 6.57 (2H, *s*, H-3' and H-5'), δ 6.01 (1H, *d*, *J* = 2.3 Hz, H-6), δ 5.91 (1H, *d*, *J* = 2.3 Hz, H-8), δ 4.81 (1H, *s*, H-2'), δ 4.18 (1H, *d*, *J* = 3.5 Hz, H-3), δ 3.52 (1H, *d*, *J* = 5.1 Hz, OH), δ 2.74 (1H, *dd*, *J* = 16.5, 4.6 Hz, H-4α), δ 2.71 (1H, *dd*, *J* = 16.5, 3.4 Hz, H-4β). The above data were identical to the literature data [44].

*cis-Tiliroside* (**17**). Yellowish powder, ^1^H-NMR (500 MHz, CD_3_COCD_3_): δ 12.37 (1H, *br s*, OH), δ 8.09 (2H, *d*, *J* = 8.8 Hz, H-2' and H-6'), δ 7.68 (2H, *d*, *J* = 8.6 Hz, H-2''' and H-6'''), δ 6.92 (2H, *d*, *J* = 8.8 Hz, H-3' and H-5'), δ 6.78 (2H, *d*, *J* = 8.6 Hz, H-3''' and H-5'''), δ 6.78 (1H, *d*, *J* = 12.9 Hz, H-7'''), δ 6.47 (1H, *br s*, H-8), δ 6.27 (1H, *br s*, H-6), δ 5.62 (1H, *d*, *J* = 12.9 Hz, H-8'''), δ 5.17 (1H, *d*, *J* = 7.3 Hz, H-1''), δ 4.28 (1H, *dd*, *J* = 11.9, 1.8 Hz, H-6β''), δ 4.17 (1H, *dd*, *J* = 11.9, 6.0 Hz, H-6α''), δ 3.55 (1H, *m*, H-2''), δ 3.55 (1H, *m*, H-5''), δ 3.49 (1H, *t*, *J* = 8.9 Hz, H-4''), δ 3.41 (1H, *d*, *J* = 9.3 Hz, H-3''). The above data were identical to the literature data [45].

*trans*-*Tiliroside* (**18**). Yellowish powder, ^1^H-NMR (500 MHz, CD_3_OD): δ 7.96 (2H, *d*, *J* = 8.8 Hz, H-2' and H-6'), δ 7.38 (1H, *d*, *J* = 16.5 Hz, H-7'''), δ 7.28 (2H, *d*, *J* = 8.5 Hz, H-2''' and H-6'''), δ 6.80 (2H, *d*, *J* = 8.8 Hz, H-3' and H-5'), δ 6.78 (2H, *d*, *J* = 8.5 Hz, H-3''' and H-5'''), δ 6.27 (1H, *br s*, H-8), δ 6.11 (1H, *br s*, H-6), δ 6.05 (1H, *d*, *J* = 16.5 Hz, H-8'''), δ 5.23 (1H, *d*, *J* = 7.4 Hz, H-1''), δ 4.31 (1H, *dd*, *J* = 11.8, 1.9 Hz, H-6β''), δ 4.18 (1H, *dd*, *J* = 11.8, 6.6 Hz, H-6α''), δ 3.55 (1H, *m*, H-5''), δ 3.48 (1H, *m*, H-3''), δ 3.47 (1H, *m*, H-2''), δ 3.46 (1H, *m*, H-4''). The above data were identical to the literature data [45].

*Isoamericanol B* (**19**). Yellowish oil, ^1^H-NMR (500 MHz, CD_3_COCD_3_): δ 7.69 (1H, *s*, OH), δ 7.06 (1H, *d*, *J* = 1.6 Hz, H-2), δ 6.92 (1H, *dd*, *J* = 8.2, 1.6 Hz, H-6), δ 6.87 (1H, *d*, *J* = 8.2 Hz, H-5), δ 6.87 (1H, *d*, *J* = 1.8 Hz, H-2'), δ 6.86 (1H, *d*, *J* = 8.2 Hz, H-5'), δ 6.79 (1H, *dd*, *J* = 8.2, 1.8 Hz, H-6'), δ 6.38 (1H, *d*, *J* = 11.8 Hz, H-7'), δ 5.76 (1H, *m*, H-8'), δ 5.18 (1H, *d*, *J* = 2.6 Hz, H-7), δ 4.60 (1H, *d*, *J* = 6.6, 2.6 Hz, H-8), δ 4.38 (2H, *d*, *J* = 5.0 Hz, H-9'), δ 3.83 (3H, *s*, OCH_3_), δ 1.68 (1H, *br s*, OH), δ 1.08 (3H, *d*, *J* = 6.6 Hz, H-9). The above data were identical to the literature data [46].

*trans*-*p*-*Coumaric acid* (**20**) White powder, ^1^H-NMR (500 MHz, CD_3_OD): δ 7.58 (1H, *d*, *J* = 15.9 Hz, H-7), δ 7.43 (2H, *d*, *J* = 8.6 Hz, H-2 and H-6), δ 6.79 (2H, *d*, *J* = 8.6 Hz, H-3 and H-5), δ 6.27 (1H, *d*, *J* = 15.9 Hz, H-8). The above data were identical to the literature data [47].

*Protocatechuic acid* (**21**) White powder, ^1^H-NMR (500 MHz, CD_3_OD): δ 7.52 (1H, *d*, *J* = 1.9 Hz, H-2), δ 7.47 (1H, *dd*, *J* = 8.3, 1.9 Hz, H-6), δ 6.89 (1H, *d*, *J* = 1.9 Hz, H-5). The above data were identical to the literature data [48].

*Salicylic acid* (**22**) White powder, ^1^H-NMR (500 MHz, CD_3_COCD_3_): δ 7.84 (1H, *d*, *J* = 7.8 Hz, H-6), δ 7.52 (1H, *t*, *J* = 7.8 Hz, H-4), δ 7.89 (1H, *d*, *J* = 8.3 Hz, H-3), δ 6.87 (1H, *t*, *J* = 8.5 Hz, H-5). The above data were identical to the literature data [49].

*trans*-*Ferulic acid* (**23**). White powder, ^1^H-NMR (500 MHz, CD_3_OD): δ 7.58 (1H, *d*, *J* = 15.9 Hz, H-7), δ 7.17 (1H, *br s*, H-2), δ 7.05 (1H, *br d*, *J* = 8.1 Hz, H-6), δ 6.80 (1H, *d*, *J* = 8.1 Hz, H-5), δ 6.30 (1H, *d*, *J* = 15.9 Hz, H-8), δ 3.88 (3H, *s*, OCH_3_). The above data were identical to the literature data [50].

*Syringic acid* (**24**). White powder, ^1^H-NMR (500 MHz, CD_3_OD): δ 7.31 (2H, *s*, H-2 and H-6), δ 3.87 (6H, *s*, OCH_3_). The above data were identical to the literature data [51].

*3-O-Methylgallic acid* (**25**) White powder, ^1^H-NMR (500 MHz, CD_3_OD): δ 7.17 (2H, *s*, H-2 and H-6), δ 3.86 (3H, *s*, OCH_3_). The above data were identical to the literature data [52].

### 3.5. Cytotoxicity Assays

#### 3.5.1. Cell Culture

Cytotoxicity was measured using the MTT assay [57]. A549 (human lung adenocarcinoma) cell line was maintained in DMEM supplemented with 10% fetal bovine serum (FBS), 100 units/mL penicillin and 100 µg/mL streptomycin (Gibco-BRL). The cells were incubated in 5% CO_2_ humidified at 37 °C for growth.

#### 3.5.2. Evaluation of Cell Proliferation by MTT Assay

The number of viable A549 cells after GA treatment was evaluated by the MTT (3-[4,5-methylthiazol-2-yl]-2,5-diphenyl-tetrazolium bromide) assay. In brief, A549 cells (3 × 10^4^ cells/well) were seeded in a 96-well plate and kept overnight for attachment. The next day the medium was replaced with fresh medium with various concentrations of EOM extract, indicated fraction (EOE, EOB, and EOW), cisplatin (positive control) and 25 compounds which were isolated from the leaves of *E. oldhamii* Maxim. (5–500 µg/mL) and cells were allowed to grow for 24 and 48 h. Four hours before completion of incubation, 10 µL of MTT (10 mg/mL) was added in each well. After completing the incubation, 100 µL of solubilization buffer (10% SDS with 0.01 N HCl) was added to each well and incubated overnight at room temperature. The optical density (OD) was measured at 450 nm using a multiscan microplate reader. Data were showed means ± SD from three independent experiments, 50% cytotoxic concentration (CC_50_) yielding 50% toxic effect was determined via a computer program (provided by John Spouge, National Center for Biotechnology Information, National Institutes of Health).

## 4. Conclusions

Twenty five compounds were isolated in the present study, including thirteen triterpenoids, five flavonoids, one lignanoid and six benzanoids compounds. Furthermore, twenty one of these compounds, including compounds **1**, **4**, **5** and **8**–**25** were obtained from *E. oldhamii* Maxim. for the first time. Furthermore, we evaluated the inhibitory activity of these compounds against the growth of non-small cell lung cancer A549 cells by MTT assay in this study. Eleven of these 25 compounds, including compounds **2**–**4**, **6**–**10**, **13**, **18** and **19** were investigated by the MTT assay for the first time regarding their cytotoxic activity in non-small cell lung cancer A549 cells. The result indicated that the five compounds, including four triterpenoids (compounds **2**, **4**, **6** and **8**) and one flavanoid (compound **18**) present in the crude extract exhibited good cytotoxic activity against the A549 cell line with CC_50_ values lower than 20 μg/mL. Furthermore, in present study these five compounds exhibited better cytotoxic activity compared to cisplatin (positive control, Table 4).

Regarding the structure-activity relationships of the triterpenoid compounds **2**, **4**, **6** and **8**, a 3-*O*-linkage to the *cis*-coumaroyl and caffeoyl moieties of oleanolic acid and ursolic acid were two active functional groups that provided better cytotoxic activity against the growth of the non-small cell lung cancer A549 cells according to the MTT assays. In addition, regarding the structure-activity relationships of the flavanoid compound **18**, a 3-*O*-linkage to D-glucose and a 6''-*O*-linkage to *cis*-coumaroyl were the active functional groups providing better cytotoxic activity against the growth of non-small cell lung cancer A549 cells in the MTT assay. These five compounds **2**, **4**, **6**, **8** and **18** may lead to the development of anti-lung cancer drugs although further studies should be performed to reveal the mechanisms of action of the active compounds found in leaves of *E. oldhamii* Maxim.
